# Two Distinct Neural Mechanisms Underlying Acupuncture Analgesia

**DOI:** 10.3389/fpain.2022.869884

**Published:** 2022-05-18

**Authors:** Yasutaka Kato, Kazuhiro Yachi, Hideyuki Hoshi, Toyoji Okada, Yoshihito Shigihara

**Affiliations:** ^1^Department of Pathology and Genetics, Hokuto Hospital, Obihiro, Japan; ^2^Acupuncture Centre, Hokuto Hospital, Obihiro, Japan; ^3^Acupuncture Clinic Kaikido, Sapporo, Japan; ^4^Precision Medicine Centre, Hokuto Hospital, Obihiro, Japan; ^5^Clinical Laboratory, Hokuto Hospital, Obihiro, Japan

**Keywords:** acupuncture, analgesia, oscillatory frequency, pain, resting-state, regional activity, magnetoencephalography

## Abstract

Acupuncture analgesia is a traditional treatment with a long history, although it lacks scientific evidence. It is reportedly associated with the central nervous system, including various brain regions, from the cortices to the brain stem. However, it remains unclear whether the distributed regions behave as a single unit or consist of multiple sub-units playing different roles. Magnetoencephalography is a neuroimaging technique that can measure the oscillatory frequency of neural signals and brain regions. The frequency band of neural signals allows further understanding of the characteristics of the acupuncture-related neural systems. This study measured resting-state brain activity using magnetoencephalography in 21 individuals with chronic pain before and after acupuncture treatment. The subjective level of pain was assessed using a visual analog scale, and brain activity was compared to identify the brain regions and the frequencies associated with acupuncture analgesia. Here, we categorized the changes in resting-state brain activity into two groups: low-frequency oscillatory activity (<3 Hz) in the left middle occipital and right superior partial lobule and high-frequency oscillatory activity (81–120 Hz) on both sides of the prefrontal, primary sensory, and right fusiform gyri. These findings suggest that acupuncture analgesia influences two or more sub-units of the neural systems, which helps us understand the neural mechanisms underlying acupuncture analgesia.

## Introduction

Pain is an unpleasant sensory and emotional experience (1) and is a major reason for clinical visits (2). Non-steroidal anti-inflammatory drugs and opiate/opioid analgesics are primarily used to relieve pain. Some alternative treatments are also available, such as manual therapy (3), transcutaneous electrical nerve stimulation, and acupuncture analgesia (4). Acupuncture analgesia is a treatment in which acupuncturists insert and manipulate fine needles at specifically designated locations on the body called acupoints to relieve pain. Acupuncture originated in China and has now become popular in Europe and America as a complementary therapy (5). It has a history of longer than 3,000 years (6, 7), although its neurological mechanism in both the peripheral and central nervous systems remains unclear.

Recently, the mechanism underlying acupuncture analgesia has become clearer through neuroimaging studies, especially in the brain. Brain activity has two major aspects: regional and non-regional. Regarding the regional aspect, “functional specialization theory” is a basic concept in human neuroscience (8). It theorizes that each brain function depends on specific brain regions: the somatic sensation is processed in the primary sensory cortex, which is situated just posterior to the central sulcus, and vision is processed in the visual cortex, which is in the occipital cortex. However, recent studies have shown that there is no “pain center”, a region specialized for pain perception, in the brain (1). Pain perception is produced by distributed regions, including the primary and secondary, somatosensory cortices, prefrontal cortices, thalamus, anterior cingulate, insular, amygdala, brain stem, and spinal cord (1). These regions are recognized as a unit called as “pain matrix” (9, 10). It is demonstrated that the matrix falls into two major sub-units playing different roles (1). One of the two sub-units is called the “lateral pain pathway,” which processes the sensory information of pain. The other unit is called the “medial pain pathway,” which processes the emotional aspect of pain.

In line with the concept of the pain matrix, acupuncture analgesia is also associated with multiple distributed regions but is not associated with a single region (i.e., “analgesia center”) in the brain. Previous studies showed that the primary and secondary somatosensory cortices (11), prefrontal and supplementary motor cortices (12), hypothalamus, amygdala (13), basal ganglia, brain stem, and cerebellum (14) play the roles for acupuncture analgesia. However, it remains unclear whether these distributed regions behave as a single unit for inducing acupuncture analgesia or consist of multiple sub-units similar to the pain matrix. For studying the behaviors of neural units distributed spatially, neuroimaging techniques must be employed to track the changes in the neural networks and signal processing. Magnetoencephalography (MEG) is an electrophysiological neuroimaging technique, which is sensitive to both regional and non-regional aspects of brain activity. The non-regional aspect is expressed in terms of spontaneous neural oscillations (15). Oscillatory frequency reflects the properties of neural activity, such as the direction of the signaling (i.e., top-down or bottom-up modulation) across the brain regions (16, 17), which have significant roles in pain regulation (18–20).

We hypothesized that if the neural changes induced by acupuncture analgesia share similar characteristics with the pain matrix, at least two types of changes would be observed, which are reflected in the oscillatory activities measured by MEG. This study aimed to categorize the neural changes relevant to acupuncture analgesia by studying the oscillatory components of resting-state brain activity measured by MEG.

## Materials and Methods

### Participants

Potential participants were recruited from the hospital staff, their friends, family members, or patients who visited our hospital for diseases other than pain and who received acupuncture treatment at Hokuto Hospital as a welfare service. To examine the present hypothesis regarding acupuncture analgesia, this study set an inclusion criterion that the participants must have severe chronic pain, which could be quantified by the visual analog scale (VAS) reading before treatment. The threshold of the VAS score was set at 50/100, and participants with pain weaker than the threshold were excluded from the analysis. All participants consented to their data being used for research and provided written informed consent. All methods were performed in accordance with the relevant guidelines and regulations in Japan. This study was conducted in accordance with the Declaration of Helsinki and was approved by the Ethics Committee of Hokuto Hospital (#1011-R2).

### Procedure

**Figure 1:**
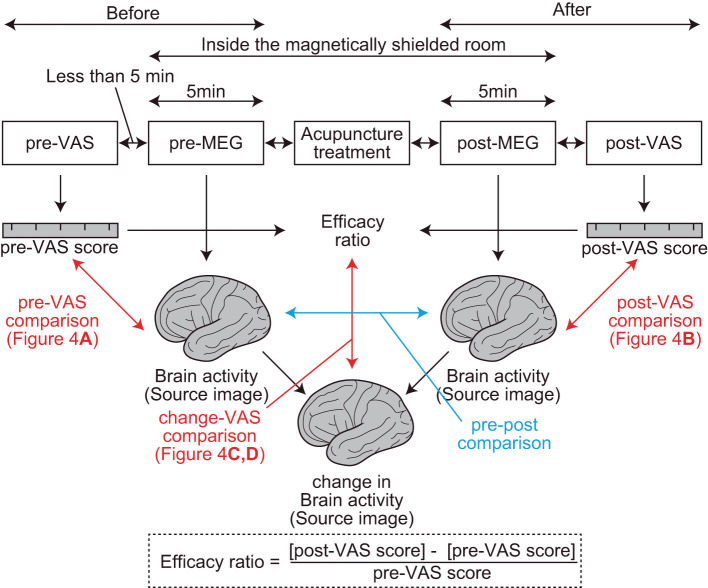
Procedures and parameters of the study. Two visual analog scale (VAS) assessments, two magnetoencephalography recordings, and one acupuncture treatment were performed sequentially (top rectangles in the figure). The interval between each step was <5 min. The red arrows represent regression analyses between the brain activity and VAS readings. The blue arrow represents a comparison between brain activities. Efficacy ratio was defined as the ratio that represents the change in the subjective level of pain before and after acupuncture treatments. Change in brain activity was calculated using the SPM function “spm_imcalc.m.”

At the beginning of the experiment, the subjective level of pain was assessed using VAS (pre-VAS) in front of the magnetically shielded room of MEG (Figure 1). During the VAS reading, participants were asked to report their “average level of pain,” which ranged from 0 (free from subjective pain) to 100 (the most severe pain ever experienced), which they experienced in their daily lives before the treatment. Then, the resting-state brain activity of the participants was recorded using MEG (pre-MEG) inside a magnetically shielded room. After the recording, participants received acupuncture treatment for pain relief by licensed acupuncturists inside the magnetically shielded room. Subsequently, resting-state brain activity was recorded again using MEG (post-MEG) without delay, followed by a second assessment for the subjective level of pain (post-VAS) in front of the magnetically shielded room. Each interval between the VAS readings, MEG scans, and acupuncture treatment was <5 min. We defined the efficiency of acupuncture analgesia as the change in VAS readings divided by pre-VAS score reading (Figure 1).

### Acupuncture Procedure

After detailed interviews regarding the participants' symptoms, an acupoint was selected by a licensed acupuncturist. The World Health Organization's standard acupuncture acupoints (21) were used for 14 participants, and other unique acupoints were used for the other seven participants (Table 1). To effectively perform the regression analysis between the efficacy ratio and resting-state brain activity, the efficacy ratio should have good variance across participants (e.g., avoid ceiling/floor effects). For this purpose, only one needle was inserted at a point somewhere in the circle (in a 2 cm radius) around the center of the acupoint. The direction and amount of acupoint deviance were arbitrarily determined by the acupuncturist. The needle was left at the acupoint for 30 s and removed promptly without rotation to elect de-qi, which is a composite of unique sensations associated with Chinese acupuncture (22). None of the participants reported that they experienced de-qi. The average depth of needling was 11.9 ± 6.3 mm (mean ± SD, range 2–33 mm).

**Table 1:** Profile of the participants and acupuncture.

**ID**	**Age**	**Sex**	**Pre-VAS (mm)**	**Post-VAS (mm)**	**Efficacy ratio**	**Chief complaint**	**Diagnoses**	**Acupoint**
1	59	F	57	52	0.09	Chronic pain in the left leg and foot	Osteoarthritis	GB33
2	41	M	52	24	0.54	Chronic low back pain	Fasciitis	BL53
3	59	F	60	10	0.83	Chronic pain in shoulder	Fasciitis	BL15
4	41	M	54	1	0.98	Chronic pain in the neck and shoulder	Cervical spondylosis	Distal hollow of the joint between the first and second metacarpal bones on the back side of the hand
5	44	F	50	3	0.94	Stiff shoulder on the right side	Cervical spondylosis	Lateral side of the spinous process of C7
6	44	F	55	11	0.80	Chronic pain in the right little finger	Thoracic outlet syndrome	Lateral side of the spinous process of C5
7	78	F	99	70	0.29	Facial pain	Trigeminal neuralgia after stroke	BL57
8	54	M	53	11	0.79	Pain in the right cheek	Cancer pain	LI4
9	35	M	70	78	−0.11	Chronic pain in the right knee	Fasciitis or Ligamentitis	Lateral side of the spinous process of T9
10	79	F	66	0	1.00	Chronic pain in the left arm	Myofascial pain	BL16
11	73	M	96	56	0.42	Chronic pain on both side of legs	Stroke	LI4
12	35	M	59	69	−0.17	Chronic pain and numbness in the right leg	Saphenous nerve entrapment	BL18
13	50	F	55	3	0.95	Chronic low back pain	Myofascial pain	BL40
14	41	F	63	33	0.48	Stiff shoulder on the right side and headache	Muscle-contraction headache and Myofascial pain	SI7
15	28	F	83	64	0.23	Abdominal pain	Menstrual pain	SP6
16	51	F	59	0	1.00	Stiff shoulder on both sides	Cervical spondylosis	Distal hollow of the joint between the second and third metatarsal bones in the dorsum of the foot
17	44	F	50	54	−0.08	Chronic low back pain	Lumber disk herniation	ST40
18	41	F	51	51	0.00	Stiff shoulder in the left	Trapezius Myalgia	BL48
19	70	F	71	19	0.73	Chronic pain in the left leg	Sciatica	DU3
20	58	F	51	49	0.04	Stiff shoulder on both sides	Cervical spondylosis	Distal hollow of the joint between the fourth and fifth metacarpal bones on the back side of the hand
21	60	F	54	50	0.07	Chronic pain and numbness in the right foot	Piriformis syndrome	ST40

*VAS, visual analog scale*.

### MEG Scanning

Resting-state brain activity was recorded for 5 min using a 160-channel whole-head type MEG system (MEG Vision; Yokogawa, Kanazawa, Japan) in a magnetically shielded room at Hokuto Hospital. During the scan, participants were in the supine position and were asked to remain calm with their eyes closed. The sampling frequency was 1,000 Hz with a 200 Hz low-pass filtering during the recording.

### MEG Analysis

**Figure 2:**
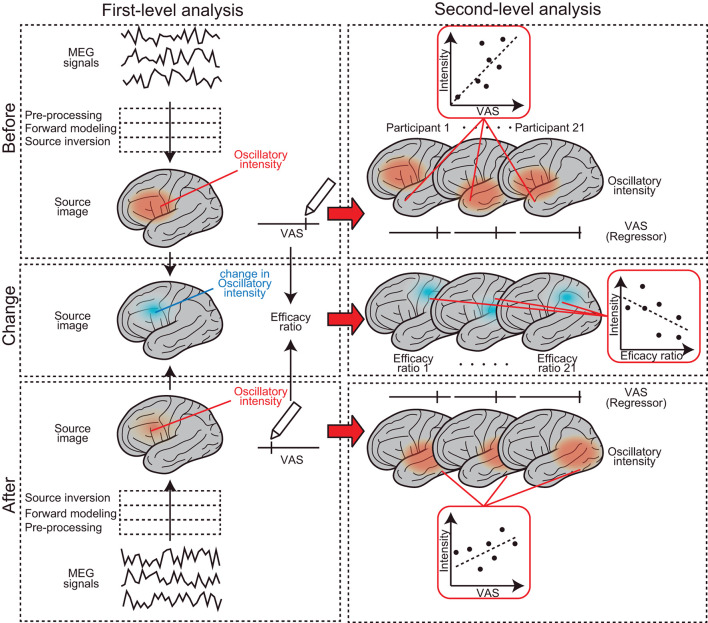
Schematic representation of the MEG analysis pipeline. It consists of two major steps: first-level (left column) and second-level analyses (right column). The first-level analysis was performed participant-wise, to gain estimated regional brain activity signals at each frequency component. Second (group)-level analysis was performed to find brain regions where the subjective levels of pain (i.e., pre-VAS, post-VAS, and efficacy ratio) were associated with regional brain activity (i.e., oscillatory intensity) at each frequency component.

The analysis protocol followed in this study was from that of our previous studies (23–25). Data were analyzed offline using SPM-12 (Wellcome Trust Center for Neuroimaging, London, UK) and the MEAW system (https://www.hokuto7.or.jp/hospital/lang/english-home/meaw/). The analysis pipeline was described elsewhere (26–28) but is illustrated schematically in Figure 2. MEG analysis consisted of two major steps: first (individual)-level and second (group)-level analyses (left and right columns in Figure 2). First-level analysis was performed participant-wise, to gain estimated regional brain activity signals at each frequency component of the delta (0–3 Hz), theta (4–7 Hz), alpha (8–12 Hz), beta (13–25 Hz), gamma (low gamma: 26–40 Hz and high gamma: 41–80 Hz), and high-frequency oscillations (HFO) (81–120 Hz). First, continuous MEG signals were divided into 10-s segments for ease of analysis (“Epoching”). Epochs in which the magnetic signal exceeded 6,000 fT were discarded (“Rejection”). A 50-Hz band-stop filter (5th order, Butterworth) was applied to the epoched data to remove artifacts from the utility frequency (“Filtering”). The three processes, epoching, rejection, and filtering, are described as “pre-processing” in Figure 2. The pre-processed data were then used for the source-level analyses. The source-level analysis included two processes, described as “forward modeling” and “source inversion” in Figure 2, which were used to identify the locations of the brain producing the resting-state-induced component. In the forward modeling, the relationships between the MEG sensors and source grids (i.e., voxels) were computed using a single-shell model with canonical magnetic resonance images provided by SPM-12. Then, the inverse estimation was made using the results of forward modeling and pre-processed data, which were performed, respectively, for seven frequency components from delta to HFO. During the source inversion, we used a maximal smoothness algorithm with a spatially coherent source model [i.e., coherence algorithms implemented in SPM-12 (abbreviated as 'COH' in SPM-12)] (29), which is comparable to the standardized low-resolution brain electromagnetic tomography method (30). Source inversions were performed by applying filters corresponding to each frequency band (from delta to HFO). Source priors were not used for source estimation. The estimated oscillatory intensity at each location was saved as a “source image” digital file in the Neuroimaging Informatics Technology Initiative format. The source images were smoothened (20 × 20 × 20 mm) and used in the second (group)-level analysis.

Second (group)-level analysis was performed to find brain regions where subjective levels of pain (i.e., pre-VAS, post-VAS, and change in VAS) were associated with regional brain activity (i.e., oscillatory intensity) at each frequency component. We used SPM-12 toolbox for the analysis, which provided a set of functions for performing quantitative statistics on the source images with technical advantages in handling multiple comparison problems that arise in the spatial dimensions of the images [i.e., family-wise error rate (FWE) corrections with 3D random field theory are available for voxel-wise hypothesis testing] (31). Four different second (group)-level analyses were performed. (1) To find brain regions in which the oscillatory intensities of the regional spontaneous neural oscillation differed before and after the acupuncture treatment (a blue arrow in Figure 1), the source images were compared between pre-MEG and post-MEG using the “paired *t*-test” option at the Factorial design specification function in SPM-12. The “paired *t*-test” is equivalent to the hypothesis testing with the null hypothesis that the binary categorical predictor has coefficient of 0 for a subjecting dependent variable (i.e., response variable), in the modeling context (32). After the model was estimated, the statistical values were visualized by building both positive (pre-MEG > post-MEG) and negative (pre-MEG < post-MEG) contrasts, which weighted the voxels, where the coefficients of the predictor have significantly deviated from zero and generated a contrast image. The voxels on the contrast image, having statistical values at *P* < 0.05 after controlling FWE across whole brains, were shown with a greyscale gradient of the statistical value (T value, in this case) on the resultant image. The resultant image was often referred to as a statistical parametric map (SPM), which is a conventional reporting style of SPM-12 (27). (2) To find the brain regions in which the oscillatory intensities of the regional spontaneous neural oscillation were predicted by the subjective levels of pain before acupuncture treatment (indicated by a red arrow in Figure 1), the source images of pre-MEG were regressed by pre-VAS reading using “Multiple regression” option at the Factorial design specification function in SPM-12. The positive and negative contrasts were examined, and SPMs were generated as the method described in (1). (3) To examine the same relationships as those in (2); however, after the acupuncture treatment, an identical analysis was performed using the post-MEG and post-VAS datasets. (4) To identify brain regions in which the changes in oscillatory intensities were predicted by the changes in the subjective levels of pain before and after the acupuncture treatment (vertical red arrow in Figure 1), an identical analysis as those in (2) and (3) was performed using the source images and VAS readings representing the changes before and after the treatment (change-MEG and the efficacy ratio for VAS). To obtain the change-MEG images, a voxel-wise algebraic subtraction (post-MEG-pre-MEG) was performed using the SPM function “spm_imcalc.m.” Cortical regions, where the peaks of the statistical values were located on the SPMs, were labeled by the default atlas in SPM-12 [i.e., atlas provided by the OASIS project (https://www.oasis-brains.org/) and Neuromorphometrics, Inc. (http://neuromorphometrics.com/)].

### Statistical Analysis

For statistical comparisons of the VAS scores, two analyses were performed. First, to examine the effect of acupuncture on VAS reading, pre-VAS and post-VAS were compared using a non-parametric bootstrapping method. Bootstrapping statistics has methodological advantages over classical statistical inferences [e.g., Gaussian assumption (33)]. The participant-wise changes between pre- and post-VAS (i.e., change-VAS) were computed. The mean of the change-VAS was computed by resampling with replacement data across all participants 10,000 times, and the ratio of the resampled average difference larger or smaller than 0 (the smaller value) was considered as the significance level (*P*-value). Second, to investigate the relationships between the participant profiles and VAS readings, a correlation analysis was performed using the bootstrapping approach. As the present study focused on acupuncture analgesia, the correlations between the efficacy ratio × pre-VAS, efficacy ratio × post-VAS, and efficacy ratio × participants' age were examined. For each pair, Pearson's coefficient (*R*) was calculated by resampling with replacement data across all participants 10,000 times. The ratio of the resampled coefficients, when larger or smaller than 0 (the smaller value), was taken as the significance level (*P*-value).

As the null hypothesis (that the bootstrapped statistics would be equal to zero) was examined repeatedly during the bootstrapping method, this series of results were at risk of an increasing Type-I error (34). To manage this risk, we reported the *P*-values controlled for the false discovery rate (FDR) using the Benjamini–Hochberg method (35). All statistical analyses were performed using the Statistics and Machine Learning Toolbox and multiple testing toolbox (36) in the MATLAB software (R2020b; The Mathworks, Inc., Natick, Massachusetts).

## Results

### Behavioral Data

**Figure 3:**
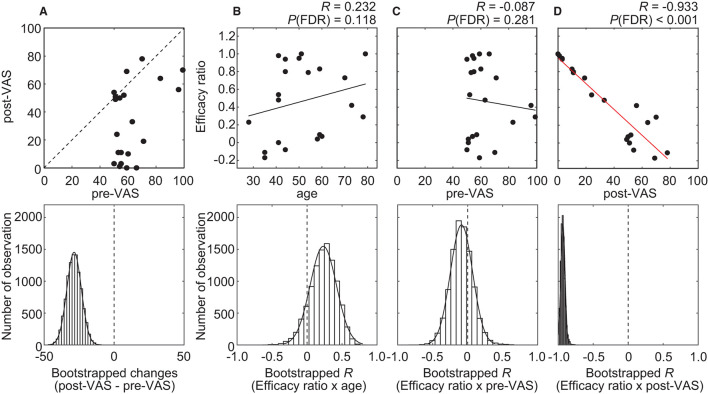
Results of the statistical analysis on behavioral data. **(A)** A scatterplot (top row) visualizes the relationship between pre- and post-VAS. The histogram (bottom row) shows the distribution of bootstrap statistics (i.e., average differences between pre- and post-VAS computed with resampled dataset) across 10,000 iterations. **(B–D)** Scatterplots (top row) and regression lines visualize the relationship between the efficacy ratio and age **(B)**, pre-VAS **(C)**, or post-VAS **(D)**. A red regression line indicates significant correlation. The histogram (bottom row) shows the distribution of bootstrap statistics [i.e., Pearson's correlation coefficient *(R)* between the efficacy ratio and age **(B)**, pre-VAS **(C)**, or post-VAS **(D)** computed with resampled dataset] across 10,000 iterations.

Altogether, 21 individuals with chronic pain participated in this study. Among all the participants, the average VAS score before and after acupuncture treatment was 62.3 ± 14.3 [mean ± standard deviation (SD)] (range, 50–99) and 33.7 ± 27.0 (mean ± SD) (range, 0–78), respectively (Figure 3A). The average level significantly decreased (*P* < 0.001) after treatment. To quantify the degree of the analgesia, the “efficacy ratio” was defined as the change in the subjective level of pain to that before treatment (Figure 1). Efficacy ratio values close to 1.0 indicated a good outcome, a value close to 0 indicated less effectiveness, and a value lower than 0 indicated increased pain. The average efficacy ratio was 0.47 ± 0.42 (mean ± SD) (range, −0.17–1.0). The efficacy ratio did not correlate with age [averaged bootstrapped *R* = 0.232; *P* (FDR) = 0.118; Figure 3B] or the subjective level of pain before acupuncture treatment [averaged bootstrapped *R* = −0.087; *P* (FDR) = 0.281; Figure 3C], while it was positively correlated with the subjective level of pain after treatment [averaged bootstrapped *R* = −0.933; *P* (FDR) < 0.001; Figure 3D].

### Comparison Between Pain and Brain Activities

**Figure 4:**
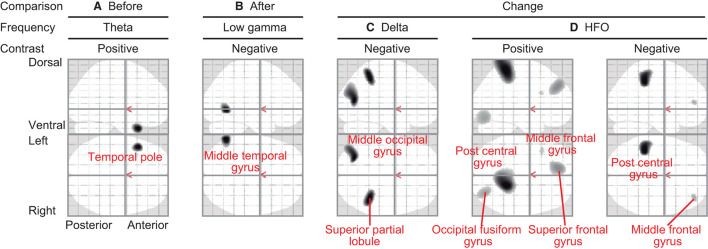
Statistical parametric maps (SPMs) reporting the locations where regional oscillatory intensities were predicted by the subjective levels of pain. **(A)** SPM of the estimated oscillatory intensity in the theta band with pre-VAS as a regressor and positive contrast (pre-VAS comparison). **(B)** SPM of the estimated oscillatory intensity in the low gamma band with post-VAS as a regressor and negative contrast (post-VAS comparison). **(C)** SPM of the estimated oscillatory intensity in the delta band with the efficacy ratio as a regressor and negative contrast (change-VAS comparison). **(D)** SPMs of the estimated oscillatory intensity in the HFO band with the efficacy ratio as a regressor and positive (left panel)/negative (right panel) contrast (change-VAS comparison).

Regional oscillatory intensities were compared with the VAS readings in three ways (red arrows in Figure 1) using regression analyses. In the pre-VAS comparison (the leftmost red arrow in Figure 1), a higher subjective level of pain predicted a stronger theta regional oscillatory intensity at the left temporal pole (Figure 4A and Table 2A). In the post-VAS comparison (the rightmost red arrow in Figure 1), a lower subjective level of pain predicted a stronger low gamma regional oscillatory intensity in the left middle temporal gyrus (Figure 4B and Table 2B). Regarding the change-VAS comparison (the red arrow in the middle in Figure 1), associations were found in two distinct frequency bands. The lower efficacy ratio predicted increasing in delta oscillatory intensity in the left middle occipital gyrus and right superior parietal lobule (Figure 4C and Table 2C). Additionally, a higher efficacy ratio predicted increasing in HFO intensities in the right postcentral, right occipital fusiform, left superior frontal, and left middle frontal gyri. The lower efficacy ratio predicted increasing in HFO intensities in the left postcentral and right middle frontal gyri (Figure 4D and Table 2D). Regarding the pre-VAS, post-VAS, and change-VAS comparisons, no significant clusters were found for the other frequency bands.

**Table 2:** Association of resting-state oscillatory intensity with the subjective level of pain and efficiency (corresponding to Figure 4).

			**Cluster-level**		**Peak-level**		**Coordinate**			
**Comparison**	**Frequency**	**Contrast**	***P* (FWE)**	**kE**	***P* (FWE)**	***T***	**X**	**Y**	**Z**	**Brain region**
(A) Pre	Theta	Positive	0.044	257	0.03	4.443	−44	18	−34	Left temporal pole
(B) Post	Low gamma	Negative	0.042	312	0.02	4.806	−58	−62	−4	Left middle temporal gyrus
(C) Change	Delta	Positive	0.039	860	1.00	2.782	−28	−72	12	Left middle occipital gyrus
					1.00	2.649	−36	−76	20	Left middle occipital gyrus
			0.042	503	1.00	2.780	40	−46	50	Right superior partial lobule
(D) Change	HFO	Positive	0.016	4,490	0.02	3.889	20	−44	62	Right post central gyrus
			0.034	874	1.00	3.032	−10	44	34	Left superior frontal gurus
			0.037	619	1.00	2.871	32	−80	−16	Right occipital fusiform gyrus
			0.048	34	1.00	2.605	−38	18	24	Left middle frontal gyrus
		Negative	0.037	583	1.00	2.805	−40	−38	44	Left post central gyrus
			0.047	49	1.00	2.573	38	46	8	Right middle frontal gyrus

*The P-values were corrected for multiple comparisons using family wise error (FWE) correction. kE, cluster size; T, t value; P, P-value; X, X-coordinate; Y, Y-coordinate; Z, Z-coordinate*.

*HFO, high-frequency oscillation (81–120 Hz)*.

Finally, the regional oscillatory intensities were compared before and after acupuncture treatment within participants (pre-post comparison; blue arrow in Figure 1) without considering the subjective level of pain. For this comparison, no significant clusters were observed in any frequency bands.

## Discussion

This study revealed that acupuncture relieved the subjective level of pain, and change in the subjective level of pain was associated with changes in the resting-state brain activities at two distinct frequency bands: delta (<3 Hz) and HFO (81–120 Hz). This supported our hypothesis that the neural changes induced by acupuncture analgesia share similar characteristics with the pain matrix, which employs two sub-units of the neural network.

Pain is not a simple perception but a complex experience affected not only by noxious stimuli but also by attention, emotion, and anticipation (1, 19, 37). This suggests that the central nervous system plays an important role in pain perception. The present study showed that the left superior frontal gyrus, both sides of the middle frontal gyri, both sides of the postcentral gyri, right superior partial lobule, right occipital fusiform gyrus, and left middle occipital gyrus contribute to pain relief (Table 2). We questioned whether these regions comprise a single system of “acupuncture analgesia” or consist of multiple sub-units similar to the pain perception system. To address this question, we used MEG to study neural activity changes induced by acupuncture analgesia, which provides information regarding the non-regional aspect of brain activity in terms of oscillatory frequencies. Oscillatory frequency is a major electrophysiological marker of non-regional brain activity. It is demonstrated that the differences in the signaling frequency would reflect the differences in the directions of signal transmission (i.e., feedback or feedforward) (19). As represented in the basic concepts of dynamic causal modeling (38) or predictive coding (39–42), neural signals in higher frequency bands tend to contribute to the sensory-driven information processing in feedforward-manner, while lower frequencies tend to do information processing across the brain regions in feedback-manner (19).

The present study discussed a highly interdisciplinary topic, which lies in the middle of nowhere between oriental medicine, neuroscience, neurophysiology, pathology, psychology, and several other fields in the humanity research (for example, the effect of acupuncture can be discussed in the context of historical study, philosophy, and sociology). In this study, we used a unique methodology, MEG measurements, which allowed us to examine the non-regional neural signaling at the whole-brain level *in vivo*. The results obtained from the electrophysiological measurements are often interpreted in the context of neuroimaging. Given the strength of this study, we discussed the results in the framework of the renowned feedback/feedforward information processing of human neuroimaging. We acknowledge that this is not an exclusive framework for interpreting the neural changes induced by acupuncture analgesia, but this would be the best approach for leveraging the strengths of the present study.

The present study identified that two distinct frequency bands were associated with the level of pain relief (i.e., efficacy ratio) (Figures 4C,D). This suggests that there are at least two distinct neural sub-units underlying acupuncture analgesia. The first sub-unit is characterized by changes in oscillatory intensities at a higher frequency band (i.e., HFO) on both sides of the postcentral gyri, middle frontal gyri, left superior frontal gyrus, and the right occipital fusiform gyrus. The postcentral gyrus covers the primary sensory cortex (43), which is responsible for processing somatosensory information and core regions of pain perception (44). It receives sensory inputs directly from nociceptors via the spinothalamic tract. Thus, it is plausible that these higher-frequency-associated regions modulate sensory-driven information processing in a feedforward-manner (39–42). This interpretation is in line with the fact that gamma and HFO activities in the primary sensory cortices are associated with subjective pain perception (45). Interestingly, the results revealed that HFO intensity in the right postcentral gyrus was positively associated with the efficacy ratio, whereas that in the left was negative. There are two possible interpretations for this finding. First, it is caused due to hemispheric asymmetry. Both sides of the cerebral hemisphere have different functions (46). Most individuals are right-handed; thus, their left hemisphere is dominant. The pain perception system is also lateralised (47–49), and the left hand is more sensitive to painful stimulation than the right (50). This asymmetry can lead to an inverse association. Second, it is caused by the interhemispheric interaction between both sides of the postcentral gyri. Although the primary sensory cortex is dominant for processing information from the contralateral side of the body, both sides of the primary sensory cortex interact with each other (43). This interaction can enhance the correlation in opposite directions.

Higher-frequency-associated regions also included the left superior frontal gyrus, both sides of the middle frontal gyrus, and the right occipital fusiform gyrus (Figure 4D and Table 2D). Although these regions may not be ordered, a previous study has reported that patients with cluster headaches demonstrated structural alternation in similar regions: the left superior, bilateral middle frontal, and fusiform gyri (51). The frontal lobe plays an important role in regulating pain processing (52, 53). The superior frontal and middle frontal gyri belong to the dorsolateral prefrontal cortex, which is associated with pain processing (52–54) and pain relief by acupuncture (12). The superior frontal gyrus is responsible for working memory (55), which is linked with attention and chronic pain (56). The middle frontal cortex plays a vital role in attention (57), which modulates pain perception (58). It is plausible that these regions contribute to acupuncture analgesia via functions of attention. The fusiform gyrus has bidirectional connectivity with the amygdala (59), which is a core structure of emotional information processing (52). The right fusiform gyrus is also associated with emotional information processing (60), which is linked to pain perception (61–63) and acupuncture analgesia (64). Emotional information processing is associated with HFO (65). Taken together, higher-frequency-associated regions modulate sensory-driven pain information directly (i.e., postcentral gyrus) or indirectly via attention (i.e., prefrontal cortex) or emotion (i.e., fusiform gyrus), and contribute to acupuncture analgesia. Notably, higher-frequency-associated regions are not consistent with either of the pain pathways (i.e., lateral or medial), and these regions could be considered as an extrinsic-information-driven subunit.

In contrast, lower-frequency-associated regions (Figure 4C and Table 2C) included the caudal part of the brain, namely the left middle occipital gyrus and the right superior partial lobule. The postcentral gyrus was not included in these regions. The neural signals in lower frequency bands tend to contribute to pain perception in a feedback-manner (19). We would suggest that these regions modulate pain perception intrinsically. The occipital region is often considered identical to the visual cortex, which is functionally specialized for visual information processing. However, our finding that the left middle occipital gyrus was associated with acupunctural pain does not conflict with the previous literature showing that acupuncture modifies the brain activity in the occipital region (66). Moreover, another study showed that brain activity in the middle occipital gyrus is associated with the efficacy of acupuncture analgesia for migraine (67), which the present finding corroborated perfectly. The middle occipital cortex plays a role in emotional information processing (68, 69). Emotional information is reflected in the delta band neural activities (70), which is potentially associated with functional cortical deafferentation or inhibition of the sensory afferences (71). The superior parietal lobule is associated with pain habituation (72) and emotional information processing (73). Taking together, we speculate that the lower-frequency-associated regions modulate inter-regional connections for pain information in feedback-manner, playing the role of an intrinsic-information-driven subunit.

Surprisingly, these regions were not identified in either the pre- or post-VAS comparison. Before treatment, theta oscillatory intensity in the left temporal pole was associated with the subjective level of pain (pre-VAS comparison; Figure 4A, and Table 2A). The temporal pole contributes to emotional information processing (74, 75), and emotion modulates pain perception (63, 76). This suggests that individual levels of pain (i.e., absolute VAS scores) were modulated by emotional factors. After treatment, the association in the temporal pole disappeared, and another association was found in the left middle temporal gyrus in the low gamma band (post-VAS comparison; Figure 4B and Table 2B). Although the function of the middle temporal gyrus is poorly known, a previous study has indicated that its activity is associated with social communication (77). Therefore, the relationship between the participants and acupuncturists may affect the subjective levels of pain after treatment. Remarkably, these findings (i.e., pre-VAS and post-VAS comparisons; Figures 4A,B) were different from those of the change-VAS comparison (Figures 4C,D). This supports the finding that the association identified in the change-VAS comparison represents brain activities associated with acupuncture analgesia.

The present study has three potential limitations. First, the causes and locations of pain and used acupoints differed because we wanted to examine the relationships between the changes in the subjective level of pain and resting-state brain activity among the general population. These random factors may have affected the results. Second, we did not consider “de-qi,” which is a unique sensation associated with Chinese acupuncture treatment. It is often used as an independent parameter and is compared with the outcome in acupuncture research (22); however, it was not considered in the present study for the following reasons: It is not commonly used in Japanese acupuncture treatments, and the strength of de-qi is subjective and difficult to replicate; thus, it is not suitable as an independent parameter, which must be controlled experimentally. Third, the present study focused only on one aspect of the acupuncture analgesic effect, i.e., the acute effect; however, the other aspect, i.e., the sustained effect, was not considered (78), which should be addressed in the future study.

In conclusion, the changes in oscillatory activities measured using resting-state MEG revealed that the neural basis of acupuncture analgesia consists of two sub-units. This is similar to the concept of pain matrix; however, the results also indicated that acupuncture analgesia induced unique changes. One sub-unit involves the regions associated with neural signals in HFO, which includes the primary sensory cortex and potentially modulates the sensory-driven pain information in a feedforward-manner. The other unit involves the regions associated with neural signals in delta oscillation and may modulate information processing across brain regions in feedback-manner via the emotional information processing system. Acupuncture does not simply interfere with afferent information but modulates the immanent system of the brain to relieve pain.

## Data Availability Statement

The original contributions presented in the study are included in the article, further inquiries can be directed to the corresponding authors.

## Ethics Statement

The studies involving human participants were reviewed and approved by this study was conducted in accordance with the Declaration of Helsinki and was approved by the Ethics Committee of Hokuto Hospital (#1011-R2). Written informed consent was obtained from all participants. The patients/participants provided their written informed consent to participate in this study.

## Author Contributions

HH and YS designed the study, analyzed the data, prepared the figures and tables, and wrote the manuscript. YK, TO, and YS operated the MEG. YK managed the project. KY practiced acupuncture treatment and supervised the other acupuncturists. All authors contributed to the article and approved the submitted version.

## Funding

This study was funded by Hokuto Hospital.

## Conflict of Interest

YS is leading a joint research project with RICOH Co., Ltd. (manufacturer of magnetoencephalography equipment). HH is now employed by RICOH Co., Ltd. The remaining authors declare that the research was conducted in the absence of any commercial or financial relationships that could be construed as a potential conflict of interest.

## Publisher's Note

All claims expressed in this article are solely those of the authors and do not necessarily represent those of their affiliated organizations, or those of the publisher, the editors and the reviewers. Any product that may be evaluated in this article, or claim that may be made by its manufacturer, is not guaranteed or endorsed by the publisher.

## Abbreviations

MEG: magnetoencephalography
VAS: visual analog scale
HFO: high-frequency oscillations
FDR: false discovery rate.
